# Potential use of mealworm frass as a fertilizer: Impact on crop growth and soil properties

**DOI:** 10.1038/s41598-020-61765-x

**Published:** 2020-03-13

**Authors:** David Houben, Guillaume Daoulas, Michel-Pierre Faucon, Anne-Maïmiti Dulaurent

**Affiliations:** 1 UniLaSalle, AGHYLE, 19, rue Pierre Waguet, 60026 Beauvais, France; 2 Ÿnsect, 1, rue Pierre Fontaine, 91000 Evry, France

**Keywords:** Agroecology, Element cycles, Biogeochemistry

## Abstract

Rearing insects is expected to dramatically increase during the next few years, and this will be associated with generating high quantities of frass (insect excreta). It is necessary to find solutions allowing the efficient valorization of these by-products before a major upscaling of the industry takes place. Therefore, this study aims at investigating the fertilizer potential of frass. A pot experiment was established and soil was amended either with mealworm (*Tenebrio molitor* L.) frass (10 Mg ha^−1^), with mineral fertilizer (NPK) at equivalent nutrient level to frass or with a mixture of 50% NPK and 50% frass. Changes of soil properties and growth and nutrient uptake by barley (*Hordeum vulgare* L.) were then analyzed. Due to its rapid mineralization and the presence of nutrient in a readily-available form, we found that frass is as efficient as mineral NPK fertilizer to improve biomass and N, P and K uptake by barley. Compared to mineral fertilizer, water soluble P concentration is five times lower in the presence of frass, which prevents P from loss and sorption onto soil constituents. More importantly, BIOLOG EcoPlate reveals that addition of frass stimulates soil microbial activity, especially when it is mixed with mineral fertilizer, suggesting a synergistic effect between both amendments. Taken together, our results indicate that frass has a great potential to be used as a partial or a complete substitute for mineral NPK fertilizer. This is especially relevant in the context of a reduced availability of mineral fertilizers while being consistent with circular economy’s principles.

## Introduction

Meeting an ever increasing food demand while reducing agriculture’s negative environmental impact is one of the greatest challenges of the twenty-first century^1,2^. Societal transition and industrial transformation are developing to ensure sustainable food production for 2.3 billion more people in the next four decades. With the demand for nutritionally crucial proteins and meat products which is expected to increase from current levels by more than 75% in 2050, we need to search for alternatives to conventional protein sources if we want to restrict agricultural land use and avoid biodiversity losses and environmental degradation^3,4^. The use of natural resources must also be reduced by adopting an eco-industrial development approach which aims at closing economic and ecological loops of resource flows^5^.

Rearing insects for mass consumption is increasingly sparking interest due to their high nutritional value and, most importantly, their resource efficiency when converting organic matter, especially waste, into protein^6–9^. As recently reviewed by Dicke^10^, insect production has a much smaller ecological footprint in terms of land and water use and greenhouse warming potential compared to the production of chicken, pigs and cattle which is related to its much lower feed to meat conversion ratio. In addition to be an alternative source of proteins for humans and animals, insect farming has also been considered to produce biofuel, some rare lipids and chitin, which has a great economical value due to its application in food, cosmetics, pharmaceuticals, textile etc.^11^.

There have been major efforts to promote the use of insects as a protein source, spurred in large part by the Food and Agriculture Organization (FAO) of the United Nations work examining the multiple dimensions of insect rearing as an important future food source and a viable option for alleviating food insecurity^12^. In European Union, novel food regulation 2015/2283 (in application since 1^st^ January 2018) clarified the legal status of insects and their derived products. Several EU members (e.g. Austria, Belgium, Denmark, Finland and Netherlands) now authorize companies to produce and sell insects as food while others (France and Germany) have partially legalized their production and commercialization^13^. These incentive policies have boosted the implementation of insect producers. For example, Ÿnsect, a French insect farming startup, has recently raised $125 million in Series C funding to develop their activities, which is the largest early-stage agtech funding deal on record in Europe (https://agfunder.com/). Overall, in EU, it is expected that the number of jobs generated by the development of this sector will increase from a few hundreds to a few thousands by 2025^14^.

Despite being highly efficient in converting biowaste into biomass, insect production itself also yields a waste stream consisting in moulting skins (exuviae) and, more importantly, insect faeces (“frass”)^10^. In natural conditions, it is well known that frass deposition to soil has a great impact on soil fertility due to its high nutrient and labile C content^15–17^. Therefore, several companies have already decided to sell frass as a fertilizer^10,18^. Even though some farmers have reported beneficial effects of frass to plants^19^, there is however currently very limited information on the ability of frass produced by insect farms to improve soil fertility and, ultimately, plant growth^20^. As stressed by Berggren *et al*.^3^, the fertilizer potential of frass needs thus urgent research before a major upscaling of the industry takes place. This would be also relevant given the urgent need to find cost-effective and environmental-friendly alternatives to conventional mineral fertilizers whose the production relies on fossil fuels and finite resources^21–23^.

Here, we evaluate the potential of frass from mealworm (*Tenebrio molitor* L.) as a fertilizer by investigating its impact on nutrient availability, soil biological activity and crop (barley; *Hordeum vulgare* L.) growth.

## Results and Discussion

### Frass characterization

**Figure 1:**
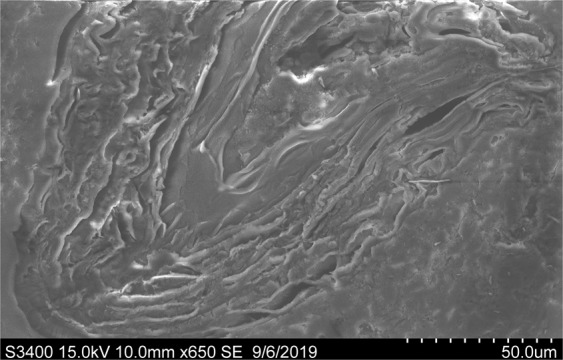
Scanning electron micrograph of frass.

**Figure 2:**
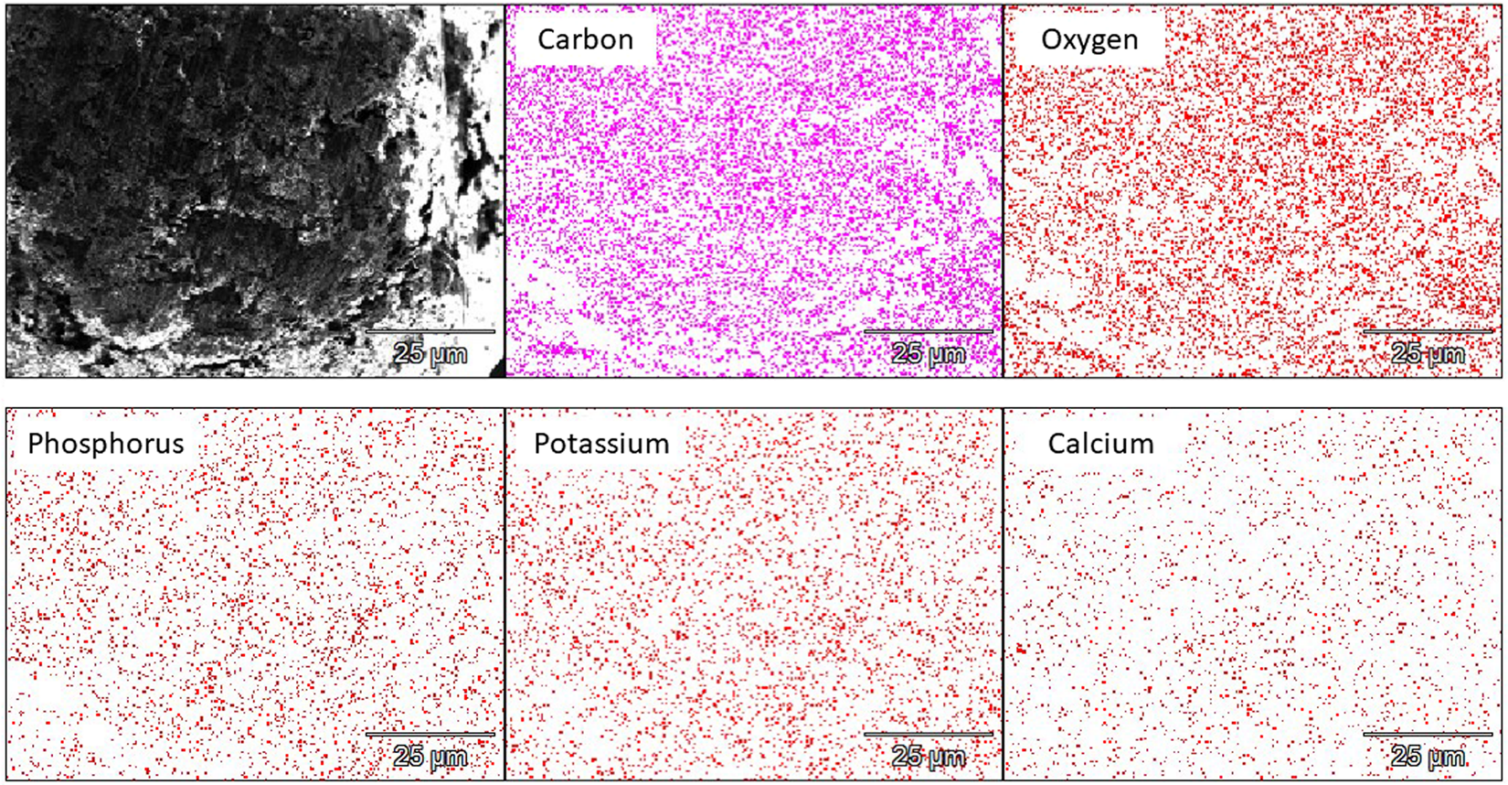
SEM-EDS analysis of the frass surface.

Chemical characterization of frass (Table 1) revealed that it had concentrations of N, K and P as high as those found in farmyard manure and, especially, poultry manure^24^, which confirms its high fertilizer potential. By contrast to conventional mineral fertilizer, frass also contained small concentrations of micronutrients (i.e. Cu and Zn), which may be further beneficial for crops. Moreover, SEM image of frass (Fig. 1) revealed that frass had a layered structure while EDS mapping (Fig. 2) showed a uniform distribution of nutrients (P, K and Ca) within the frass organic matter (represented by the C and O maps), suggesting the absence of isolated mineral phases which might potentially drive nutrient release by frass. Thus, the release of nutrients by frass after its incorporation into soil should be homogenous and possibly more extended than fertilizers in which nutrients are unevenly distributed^25^. The C fractionation in frass (Table 1) was also similar to that of poultry manure^26^, with high soluble C fraction and low cellulose-like and lignin-like fractions. This high fraction of labile C is in agreement with previous findings and may be related to the high digestion of cellulose and lignin compounds by mealworm larvae^27,28^.

**Table 1:** Chemical characteristics of frass.

Organic C g kg^−1^	Total N g kg^−1^	Total K g kg^−1^	Total P g kg^−1^	Total Cu mg kg^−1^	Total Zn mg kg^−1^
393	50	17	20	10	94.2
pH	EC dS m^−1^	Soluble fraction %Corg	Hemicellulose-like fraction %Corg	Cellulose-like fraction %Corg	Lignin-like fraction %Corg
5.8	5.3	49.3	31	15.2	4.4

### Frass mineralization

**Figure 3:**
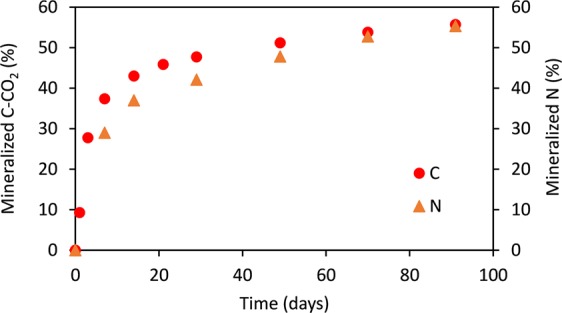
Carbon and nitrogen mineralization dynamics of frass.

In line with results from natural ecosystems showing that insect frass was rapidly decomposed in an early decomposition process^17,29^, our data showed that frass from mealworm farm was quickly mineralized after its incorporation into the soil (Fig. 3). After 7 days of incubation, 37% of total organic carbon (TOC) was mineralized. Then, a slower but continuous C mineralization occurred, reaching 56% of TOC after 91 days. Nitrogen mineralization had a similar pattern: a rapid mineralization just after frass application (37% of total N after 17 days of incubation) was followed by a slower but continuous N mineralization (55% of total N at the end of the incubation period). In terms of mineralization, the mealworm frass used in this study compares well with frass from other insects. For instance, Lovett and Ruesink^17^ reported that gypsy moth frass was highly decomposed in a 120-day incubation experiment, with the greatest mineralization in the first 10 days. The mealworm frass used in this study had a high labile C concentration (Table 1) and this likely stimulated microbial growth, resulting in high rate of C and N mineralization^16,30,31^. This is in agreement with Rothé *et al*.^26^ who found that the mineralization pattern of poultry manure, which had a similar chemical composition than frass, was intense in the early stages of incubation due to its high labile C concentration.

### Microbial metabolic activity and diversity

**Figure 4:**
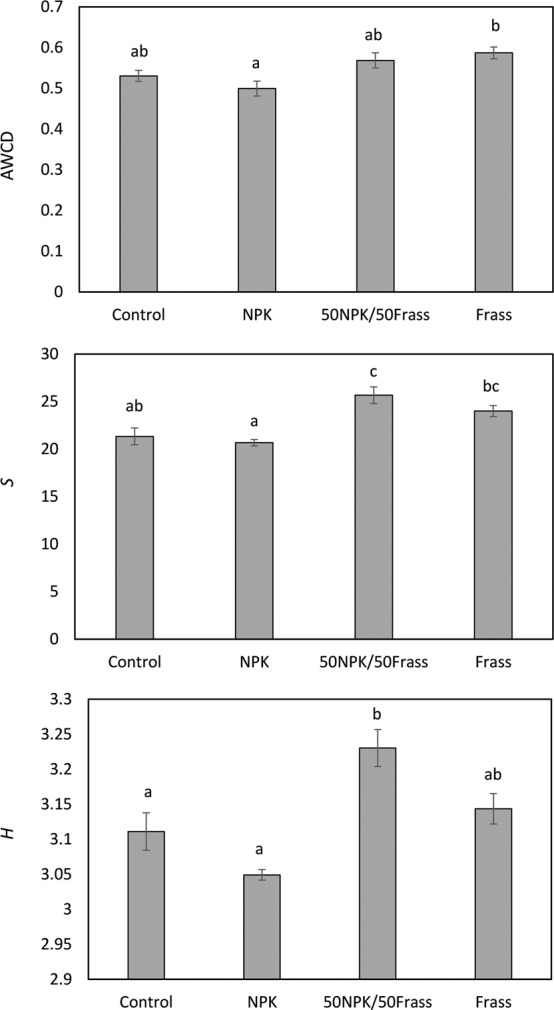
Average well-color development (AWCD), Richness (*S*) and Shannon-Weaver index (*H*) of metabolized substrates in BIOLOG EcoPlate after frass application. Values are average (n = 3) ± standard error. Columns with same letter do not differ significantly at the 5% level.

Microbial metabolic activity and diversity were assessed through the measurement of average well color development (AWCD) and *S* and *H* indices in BIOLOG EcoPlate (Fig. 4). AWCD reflects the oxidative capacity of soil microorganisms and may be used as an indicator of soil microbial activity^32^. Organic amendments are source of available energy for soil microorganisms. As a result, the supply of organic matter to soil through different amendments generally stimulates microbial activity and increases the microbial community functional potential, which can be reflected by an increase of AWCD and *R* and *H* indices^32^. Similar to other organic amendments, the incorporation of frass, either at 50% or 100%, resulted in an increase of the AWCD and/or *S* indices compared to the NPK treatment. These results are consistent with Zhong *et al*.^33^ who reported that AWCD and diversity indices were increased by the application of organic manure alone or with NPK compared to the NPK applied alone, and reflects that the addition of organic C, alone or in combination with NPK, led to C utilization pattern shifts and increased soil microbial functional diversity. Compared to the control, the lack of improvement of *S* index in the frass treatment might be explained by the relatively low rate of frass application in the present study (i.e. 10 Mg ha^−1^). Indeed, Gomez *et al*.^32^ showed that, although organic amendments increased AWCD at both 10 and 20 Mg ha^−1^ application rate, *S* index was only improved in the 20 Mg ha^−1^ treatment due to higher C availability for microorganisms. However, *S* and *H* indices were interestingly the highest in the 50NPK/50Frass treatment. According to Hu *et al*.^34^, higher diversity in soil amended with a combination of both organic and mineral fertilizers is due to a larger number of C sources under this treatment: the presence of the organic amendment provided direct C sources while the addition of NPK provided indirect C sources by stimulating the release of root exudates. Since the functioning of ecosystems strongly relies on soil biodiversity^35,36^, the added benefits resulting from the combined use of frass and NPK on soil microbial functional diversity suggests that a partial substitution of mineral fertilizer by frass could be an enticing option for the sustainable management of agroecosystems. It must however be noted that our study only focused on soil microorganisms. The next challenge will be therefore to investigate the effect of frass on soil fauna as it also plays a key role on the regulation of ecosystem functioning^37^.

### Soil properties

**Figure 5:**
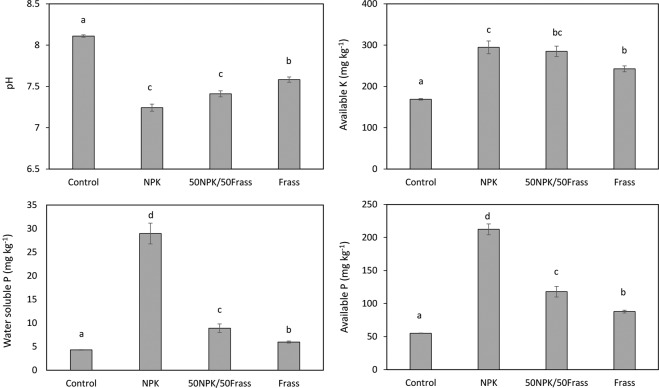
pH and concentrations of water soluble P and available K and P in soil. Values are average (n = 4) ± standard error. Columns with same letter do not differ significantly at the 5% level.

Although the soil pH of the frass treatment was not as low as that of the NPK treatment, the addition of frass significantly decreased soil pH, most likely due to the slightly acidic nature of frass (Table 1) as well as to its rapid decomposition which led to the production of CO_2_ and organic acids (Fig. 5). The fast nitrification, as reported in the incubation experiment (Fig. 3), may be an additional source of protons in the frass treatment^38^. Even though all the fertilizer treatments added the same total amounts of P and K, the highest available K and P concentrations were measured in the NPK treatment which was obviously due to the supply of these nutrients in a soluble form (Fig. 5). However, available K and P concentrations were greater in the frass treatment than in the control, suggesting that frass provided nutrients in a readily available form. The lower K and P concentrations in the presence of frass compared to the NPK treatment is in agreement with other studies comparing organic and mineral fertilizer over similar short experimental duration^39^. For instance, Houben *et al*.^22^ found that, after 100 days, available P concentration in a soil amended with sewage sludge was 50% lower than that in the presence of a mineral fertilizer. However, after one year, the authors found that both sewage sludge and mineral fertilizers were equally effective to improve P availability due to the continuous mineralization of sludge. Compared to mineral fertilizer, Andrews *et al*.^40^ found that compost increased K availability by 11 and 20% after 1 and 3 years, respectively, which was attributed to the K supply by compost and the increase of exchange sites due to organic matter added. These findings stress the need for longer-term experiment to better constrain the kinetics of nutrient release by frass. The available K and P concentrations when 50% of the NPK were substituted by 50% frass (50NPK/50Frass treatment) were similar and lower, respectively, than when NPK is used alone but higher compared to the complete NPK substitution by frass. This suggests that available K and P concentration in the 50NPK/50Frass treatment reflects both the rapid K and P supply by the conventional fertilizer and the slightly slower K and P supply by frass, as previously found for other organic amendments^41^. In addition, the supply of soluble organic carbon by frass might improve P availability by reducing sorption of P supplied by mineral fertilizer due to complexation with Fe, Al and Ca, which prevents P precipitation, and competition with inorganic P for the same sorption sites^42^. On the other hand, P and K supply by mineral fertilizer may promote the release of P and K from frass by stimulating its mineralization^43^, which is consistent with the elevated microbial metabolic activity and diversity shown by this treatment.

### Biomass and nutrient uptake

**Figure 6:**
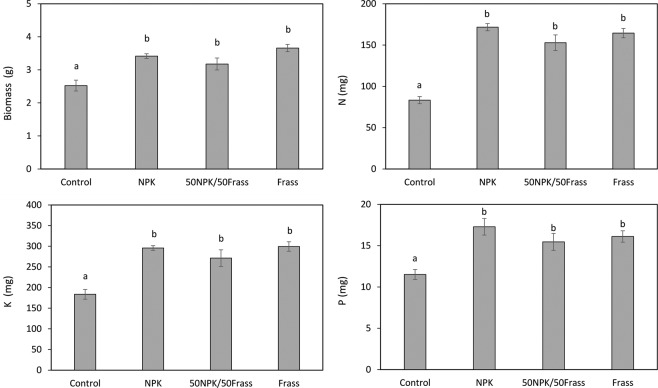
Biomass and concentrations of N, K and P of harvested barley. Values are average (n = 4) ± standard error. Columns with same letter do not differ significantly at the 5% level.

Despite having lower available P or K and P concentrations, the 50NPK/50Frass and frass treatment, respectively, were as effective as the NPK treatment to improve barley biomass and nutrient uptake (Fig. 6), indicating a high effectiveness of N, K and P contained in frass. This may be explained by the rapid frass mineralization and the presence of N, K and P in a readily available form which gradually supplied nutrient to plants throughout the growing period^44^. It is also likely that barley was able to mobilize nutrients from less available pool, as demonstrated by Nobile *et al*.^23^ who reported that, due to high specific root length and great carboxylate release, barley acquired as much P from sludge as from mineral fertilizer.

Compared to sole application of either NPK or frass, the combination of half doses of both did not significantly impact plant biomass and nutrient uptake, suggesting no interaction effects between both amendments (Fig. 6). Mixing organic amendment with mineral fertilizer is known to improve plant growth because organic matter help retaining soluble nutrients added by mineral fertilizer, preventing them from leaching^45^. Under the current pot experiment conditions where irrigation is controlled, the possibility of nutrients losses via leaching is very low and this most likely explained the lack of synergistic effect between frass and NPK^46^. In field experiment should therefore be carried out to confirm the ability of frass to reduce leaching of nutrients released by mineral fertilizer. On the other hand, the similar effect of frass on biomass and nutrient uptake as compared to NPK suggests that frass acted as a nutrient source and could be used as a partial or complete substitute of mineral fertilizer. Interestingly, despite having much lower water soluble P concentrations (Fig. 5), treatments with frass induced similar P uptake than the NPK treatment (Fig. 6). Water soluble P is known to be closely related to P loss in runoff and leaching waters^47,48^ and is therefore considered as an important index for soil P status in the environment^49^. On the other hand, water soluble P in NPK can be rapidly fixed in less available forms in the soil due to strong sorption on soil constituents^50^. Since P uptake by plants was not impaired by the lower water soluble P concentration in frass treatments, it appears that P in frass may be mineralized and released during the incubation and became available for plant uptake and/or be protected by organic substances from sorption to sparingly available forms, as reported for other organic amendments^51^. Thus, by limiting water soluble P concentration without impacting P uptake by plants, frass prevents P from loss and sorption while acting as an effective fertilizer. One perspective of this work will be to test the effect of frass on the growth and nutrient uptake of other crop species in order to determine to what extent the ability of frass to supply high amount of nutrients to plants is mediated by the strategies of plants to mobilize and acquire these nutrients.

## Conclusion

Insect production is expected to dramatically grow in the next few years due to the increasing need of finding alternative sources of protein. Given the « zero waste » context and the need to contribute to the circular economy, it is necessary to capitalize on all components of the insects, including their frass. This study indicates that frass has a great potential to be used as a partial or a complete substitute of mineral NPK fertilizer. Indeed, due to its rapid mineralization and its high content in readily-available nutrient, frass has similar effectiveness to supply N, P and K and sustain biomass production than NPK fertilizer. In addition, compared to mineral fertilizer, water soluble P concentration is up to five times lower in the presence of frass, which prevents P from loss and sorption onto soil constituents. Most importantly, the presence of frass may increase microbial metabolic activity and diversity, suggesting a better soil functioning, especially when frass is combined with mineral fertilizer.

As this was a greenhouse study, further *in situ* researches are required because temporal mineralization in controlled conditions may be different from mineralization in field due to e.g. differences of moisture, temperature and soil and crop biodiversity, thereby affecting possibly the temporal release of nutrient for plants. Nonetheless, our findings suggest that the forecasted growing amount of frass generated in the near future might constitute a sustainable resource for managing NPK nutrition in cropping system and a promising alternative to conventional fertilizer.

## Materials and methods

### Frass

Frass from mealworm (*Tenebrio molitor* L.) was provided in the form of powder by Ÿnsect (ŸnFrass, Paris, France), an industrial company farming this insect at the large-scale. The mealworms were fed exclusively on local agricultural raw materials (wheat bran) authorized by French and European regulations for farm animal feeds. The frass was hygienized (70 °C, 60 minutes) and used without any chemical input, making it a fertilizer compatible with organic farming and not subject to any specific restrictions. Moisture content was 12.4% (103 °C; 4 h). Frass was analyzed for pH and electrical conductivity in a 1:5 frass: water suspension (w/v), organic C content^52^, total nitrogen (N) content^53^, phosphorus (P), potassium (K), copper (Cu) and zinc (Zn) concentrations^54^. Four biochemical fractions were determined using a modified Van Soest method^55^: soluble (SOL), hemicellulose-like (HCE), cellulose-like (CEL) and lignin-like (LIG).

### SEM-EDS characterization of frass

The inner morphology of frass was investigated on polished section of frass particles impregnated with epoxy using a scanning electron microscope (SEM; Hitachi S3400N). The SEM was equipped with an energy-dispersive X-ray spectrometer (EDS; Thermo Ultradry) for element detection and element mapping was carried out on frass particles mounted on double-sided adhesive carbon tape. Voltage was set at 20 kV and counting time was 200 seconds.

### Soil

The study soil was sampled in Beauvais (Northern France) and was classified as a Haplic Luvisol^56^. A total mass of 100 kg was obtained by composite sampling (0–10 cm depth) from a cultivated land. After sampling, the soil was air-dried, crushed and sieved at 2 mm. Particle size analysis using the pipette method^57^ revealed that the soil was a silt loam (USDA classification) with 16% sand, 67% silt, and 17% clay. Chemical characterization of the soil was carried out using the procedures described in Houben *et al*.^58^ and revealed that organic C was 1.54%, total N was 0.18%, the cation exchange capacity (CEC) was 12.5 cmol_c_ kg^−1^ and pH was 7.8. Available concentrations as assessed using the acetate ammonium-EDTA extraction^58^ were Ca 3869 mg kg^−1^, Mg 101 mg kg^−1^, K 292 mg kg^−1^, P 72 mg kg^−1^.

### Description of treatments

The frass was applied to the soil at a rate of 10 Mg dry matter ha^−1^ (hereafter called “Frass” treatment). A mineral control adding the same quantity of N, P and K as in the Frass treatment was achieved by mixing the soil with appropriate amount of inorganic nutrients (NH_4_NO_3_, KH_2_PO_4_ and KCl) in solution (hereafter called “NPK”). Untreated soil (hereafter called “control”) and another treatment adding 50% frass plus 50% NPK were also performed (hereafter called “50NPK/50Frass”).

### Incubation experiment: C and N mineralization

The kinetics of frass mineralization were followed during laboratory incubations of control and frass treatments, based on the French normalization^59^. An amount equivalent to 25 g of dry soil mixture was incubated in 1.2 L hermetic jars kept in a dark room at 28 °C. The experiment was conducted in four replicates and lasted 91 days. The water content of the mixtures was adjusted at field capacity with demineralized water and controlled during the incubation period. In each glass jar, C mineralized as CO_2_ was trapped in 10 mL of 0.5 mol L^−1^ NaOH, which was replaced after 1, 3, 7, 14, 21, 28, 49, 70 and 91 days of incubation. The C-CO_2_ trapped in NaOH was determined by titration of the residual NaOH after precipitation of the carbonate with barium chloride. Similar incubation was carried out for N mineralization. Mineral N (N-NH_4_^+^ and N-NO_3_^−^) was extracted by shaking the mixtures for 1 h with 100 mL 1 mol L^−1^ KCl after 0, 7, 14, 28, 56 and 91 days of incubation^60^. The mineral N in the extracts was analysed by colorimetry on a continuous flow analyser (Skalar, The Netherlands). As recommended by Doublet *et al*.^60^, the dynamics of C mineralisation in soil was calculated by subtracting C-CO_2_ mineralised in the control treatment to C-CO_2_ mineralised from the frass treatment and the results were expressed as a percentage of the total organic C (TOC) in frass. The release of mineral N from frass was calculated by subtracting the mineral N measured in the control treatment from the mineral N in the frass treatment.

### Pot experiment

A pot experiment was conducted to determine the effect of frass on the nutrient availability for plants. Plastic plant pots were filled with 3500 g of each mixture in four replicates. Before sowing, th pots were placed in a controlled dark room and the mixtures were equilibrated during two weeks at 80% water holding capacity. After the equilibration period, the pots were transferred to a greenhouse glass and were arranged according to a randomized design. In each pot, 8 seeds of barley (*Hordeum vulgare* L.) were sown. After 10 days, the plants were thinned to two plants per pot. The trials were conducted under controlled greenhouse conditions (temperature 18–25 °C, 16 h photoperiod) with daily sprinkler watering to maintain the soil moisture at field capacity. After 9 weeks, shoots were harvested with ceramic scissors, dried (60 °C; 72 h), weighed and crushed. The content of P and K in aerial parts was analysed by inductively coupled plasma-atomic emission spectroscopy (ICP-AES; Jarrell Ash) after *aqua regia* digestion. The content of N in aerial parts was analysed using the Dumas combustion method.

### Soil analyses

After the harvest, water-soluble phosphorus concentration in soil (soil:water 1:60; w-v) was determined as described by Sissingh^61^. The available K and P concentrations were determined using the ammonium acetate-EDTA soil test^58^. Soil pH was measured in H_2_O (soil:water 1:5; w-v). Community-level physiological profiles (CLPPs) based on the ability of microorganisms to oxidise various substrates were assessed using BIOLOG EcoPlates (BIOLOG Co., Hayward, USA). Each 96-well plate consisted of three replicates, each one comprising 31 sole C sources and a water blank. Five grams of soil collected at the end of the pot experiment, were shaken with 45 ml of sterile 0.85% NaCl for 30 min at 200 rpm and then diluted to 1:1000. Each plate well was inoculated with 150 µL of the dilution and the plates were incu bated at 25 °C. Color development for each well was obtained in terms of optical density (OD) at 590 nm using an automated plate reader. Average well colour development (AWCD) was calculated as a measure of microbial functional diversity. AWCD was calculated as:

$${\rm{AWCD}}=\sum ({{\rm{C}}}_{{\rm{i}}}-{\rm{R}})/31$$

where R is the absorbance of the control well (containing water instead of C source) and C_i_ is the absorbance of plate well inoculated with C source i. Richness (*S*), as the number of oxidized C substrates and the Shannon-Weaver index (H) were calculated using an OD of 0.25 as threshold for positive response^32^. Shannon-Weaver index was calculated as follows:

$${\rm{H}}=-\,\sum {p}_{i}(\mathrm{ln}\,{p}_{i})$$

where

$${p}_{i}=({{\rm{C}}}_{{\rm{i}}}-{\rm{R}})/\sum ({{\rm{C}}}_{{\rm{i}}}\mbox{--}{\rm{R}}).$$

Kinetic curves suggested that after 72 h incubation time, the wells with the most active microbial communities reached the asymptote of color development. Therefore, this point was considered as the optimal incubation time for further statistical analyses, as suggested by Doan *et al*.^62^.

### Statistical analyses

All recorded data were analysed using descriptive statistics (mean ± standard error) and normality was determined using the Shapiro-Wilk test. The data were subjected to one-way ANOVA and Tukey’s post-hoc test to compare treatments, which had a normal distribution. Data without normal distribution were subjected to the Kruskall-Wallis test and Mann-Whitney post-hoc test. All statistical analyses were performed using R software version 3.5.0^63^ and the package Rcmdr^64^.
